# Integrative Transcriptomic, Lipidomic, and Metabolomic Analysis Reveals Potential Biomarkers of Basal and Luminal Muscle Invasive Bladder Cancer Subtypes

**DOI:** 10.3389/fgene.2021.695662

**Published:** 2021-08-16

**Authors:** Chao Feng, Lixin Pan, Shaomei Tang, Liangyu He, Xi Wang, Yuting Tao, Yuanliang Xie, Zhiyong Lai, Zhong Tang, Qiuyan Wang, Tianyu Li

**Affiliations:** ^1^Institute of Urology and Nephrology, The First Affiliated Hospital of Guangxi Medical University, Nanning, China; ^2^Center for Genomic and Personalized Medicine, Guangxi Medical University, Nanning, Nanning, China; ^3^Department of Urology, The First Affiliated Hospital of Guangxi Medical University, Nanning, China; ^4^Guangxi Key Laboratory for Genomic and Personalized Medicine, Guangxi Collaborative Innovation Center for Genomic and Personalized Medicine, Nanning, China; ^5^Department of Biochemistry and Molecular Biology, School of Basic Medical Sciences, Guangxi Medical University, Nanning, China; ^6^School of Information and Management, Guangxi Medical University, Nanning, China; ^7^Department of Gastroenterology, The First Affiliated Hospital of Guangxi Medical University, Nanning, China; ^8^Department of Urology, Affiliated Tumor Hospital of Guangxi Medical University, Nanning, China

**Keywords:** MIBC, subtype, transcriptomic, lipidomics, metabolomic

## Abstract

Muscle invasive bladder cancer (MIBC) is a heterogeneous disease with a high recurrence rate and poor clinical outcomes. Molecular subtype provides a new framework for the study of MIBC heterogeneity. Clinically, MIBC can be classified as basal and luminal subtypes; they display different clinical and pathological characteristics, but the molecular mechanism is still unclear. Lipidomic and metabolomic molecules have recently been considered to play an important role in the genesis and development of tumors, especially as potential biomarkers. Their different expression profiles in basal and luminal subtypes provide clues for the molecular mechanism of basal and luminal subtypes and the discovery of new biomarkers. Herein, we stratified MIBC patients into basal and luminal subtypes using a MIBC classifier based on transcriptome expression profiles. We qualitatively and quantitatively analyzed the lipids and metabolites of basal and luminal MIBC subtypes and identified their differential lipid and metabolite profiles. Our results suggest that free fatty acids (FFAs) and sulfatides (SLs), which are closely associated with immune and stromal cell types, can contribute to the diagnosis of basal and luminal subtypes of MIBC. Moreover, we showed that glycerophosphocholine (GCP)/imidazoles and nucleosides/imidazoles ratios can accurately distinguish the basal and luminal tumors. Overall, by integrating transcriptomic, lipidomic, and metabolomic data, our study reveals specific biomarkers to differentially diagnose basal and luminal MIBC subtypes and may provide a basis for precision therapy of MIBC.

## Introduction

Bladder cancer (BC) is the 10th most common malignancy worldwide (Bray et al., 2018). BC can be classified into non-muscle-invasive bladder cancer (NMIBC) and muscle-invasive bladder cancer (MIBC) based on the depth of tumor cells invasion (Kamat et al., 2016). Approximately 25% of BC patients are diagnosed with MIBC, which has a higher rate of relapse and worse prognosis than NMBIC. Neoadjuvant cisplatin-based chemotherapy (NAC) before radical cystectomy is the standard treatment option for MIBC patients (Grossman et al., 2003; International Collaboration of Trialists et al., 2011). However, approximately 40% of MIBC patients benefit from NAC, and only a minority of patients with MIBC respond to immunotherapy (Zargar et al., 2015). Therefore, new MIBC diagnostic biomarkers and therapeutic strategies are urgently needed.

Accumulating evidence indicates that MIBC is a heterogeneous disease that can be divided into different molecular subtypes based on transcriptome profiles or specific genomic alterations (Sjodahl et al., 2012; Cancer Genome Atlas Research Network, 2014; Robertson et al., 2017; McConkey and Choi, 2018). MIBC can be grouped into basal and luminal subtypes with distinct classifiers or models, which are similar to the molecular subtypes used to stratify types of breast cancer (Damrauer et al., 2014; Sjodahl et al., 2017). Among these classifiers, a clinically significant panel of 47 genes (BASE47) is used as a classifier of high-grade MIBC. BASE47 accurately discriminates intrinsic MIBC subtypes and promotes an understanding of MIBC pathobiology (Damrauer et al., 2014). Typical urothelial basal cells markers, such as KRT6B, KRT14, and KRT5, are highly expressed in basal tumors, while luminal tumors express high levels of genes that mark terminal urothelial differentiation, such as those seen in umbrella cells (KRT20, UPK1B, UPK3A, and UPK2). MIBC subtypes not only demonstrate distinctive biological characteristics but also have prognostic and therapeutic value. Basal MIBC has a worse prognosis and a higher rate of metastasis than the luminal subtype (Choi et al., 2014a; Robertson et al., 2017). Moreover, basal MIBC subtype is more sensitive to anti-epidermal growth factor receptor (anti-EGFR) agents and cisplatin-based combination chemotherapy than the luminal subtype (Choi et al., 2014a,b). Given the complex heterogeneity of MIBC, there is an urgent need for the definition of subtype-specific biomarkers that can be applied for more precise management and therapeutic interventions for MIBC.

The reprogramming of metabolic patterns in tumor tissue facilitates the rapid proliferation of tumor cells in the absence of oxygen and nutrients and drives tumor progression (Putluri et al., 2011; Nuhn et al., 2012). The tumor metabolome originates from the interaction of genome, transcriptome, proteome, and a series of external influences. Metabolomic signatures mirror the dynamic biochemical activity of the tumor’s pathobiology (Loras et al., 2018). Therefore, over the last decade, research had increasingly focused on the identification of novel biomarkers associated with metabolomics for the early detection of cancer (Alberice et al., 2013; Frantzi et al., 2016; Yumba Mpanga et al., 2018). Although previous research had concentrated on BC metabolism for screening and detection (Sahu et al., 2017), it has become evident that lipid metabolism is also an important component to be considered. Lipids are employed to store energy; they are also involved in cell membrane synthesis and act as messengers for molecular recognition and signal transduction (Larrouy-Maumus, 2019). Lipid metabolism is closely related to cancer progression (Munir et al., 2019). Thus, both lipidomics and metabolomics play vital roles in the occurrence and development of cancer. However, to date, differential lipid and metabolite profiles between basal and luminal MIBC subtypes have not been examined.

Herein, we integrated transcriptomic, lipidomic, and metabolomic analyses to identify the differential lipids and metabolites between basal and luminal MIBC subtypes, which will provide potential biomarkers for precision therapy of MIBC.

## Materials and Methods

### Clinical Samples

The 12 MIBC tissues used in this study were obtained from The First Affiliated Hospital of Guangxi Medical University in China from June 2019 to June 2020. Patients undergoing chemotherapy or radiotherapy before surgical resection were excluded, and the diagnosis of MIBC was confirmed by two experienced pathologists.

### RNA Sequencing

Total RNA was extracted from tissues using TRIzol^®^ reagent (Invitrogen, Carlsbad, CA, United States) according to the manufacturer’s protocol. Ribosomal RNA (rRNA) was removed from the total RNA using Ribo-Zero rRNA removal kits (Illumina, San Diego, CA, United States). Complementary DNA (cDNA) libraries were constructed by reverse transcription of the purified messenger RNAs (mRNAs). The libraries were amplified by PCR, followed by sequencing for 150 cycles on an Illumina HiSeq 4000 sequencer (Illumina). The quality of the raw sequencing data was assessed using FastQC software. Fastp was used to preprocess the raw data (Chen et al., 2018). The clean data were mapped to the human genome (hg19) using HISAT2 (Kim et al., 2019) and StringTie (Pertea et al., 2015, 2016), and Cufflinks was used to merge the data (Ghosh and Chan, 2016). The 47-gene panel was used to accurately separate MIBC samples into luminal and basal subtypes (Damrauer et al., 2014). Gene set enrichment analysis (GSEA) was conducted based on the default parameters, using mRNA expression profiles of samples. The xCell algorithm was used to specifically infer 64 immune and stromal cell types in each sample, based on mRNA expression profiles (Aran et al., 2017). The expression profiles of samples were prepared and uploaded to the xCell web^1^. Analysis was performed by xCell signature (*N* = 64) with 1,000 permutations, based on the parameter settings.

### Tissue Metabolome Extraction

Extraction methods were performed as previously reported (Yuan et al., 2012). Tissues were ground using the Precellys evolution system (Bertin Technologies, Saint Quentin en Yvelines, French) under 1,600 × *g*, for 10 s, two cycles, and a 5-s pause. Samples were then incubated at 500 × *g* for 30 min at 4°C. The sample was centrifuged at 4,000 × *g* for 10 min at 4°C; then, the supernatant was isolated and dried by Genevac miVac (Tegent Scientific Ltd., Ipswich, United Kingdom). Precipitates were resuspended in 100 μl of 1% acetonitrile, and the supernatant was isolated for further analysis.

### Metabonomics Data Acquisition

Ultrahigh performance liquid chromatography (UPLC, Agilent 1290 II, Agilent Technologies, Waldbronn, Germany) combined with tandem quadrupole time-of-flight (5600 Triple TOF Plus, AB Sciex, Singapore), and ACQUITY UPLC HSS T3 (1.8 μm, 2.1 mm × 100 mm, Waters, Dublin, Ireland) chromatographic column were used for the analysis. All analyses were performed in electrospray ionization mode. Instrument conditions were as previously reported (Song et al., 2020), including the following: curtain gas = 35; positive ion spray voltage = 5,500 V; negative ion spray voltage = -4,500 V; temperature = 450°C; ion source gas 1 = 50; and ion source gas 2 = 50. Data acquisition mode included a full scan of the primary mass spectrum and information-dependent acquisition of secondary mass spectrum data. MarkerView 1.3 (AB Sciex, Concord, ON, Canada) was used to extract the peak area, mass-to-charge ratio, and retention time of the primary mass spectrum data to generate a two-dimensional data array. Secondary mass spectrum data were extracted by PeakView 2.2 (AB Sciex), and metabolite IDs were identified after interrogation of a metabolite database, HMDB, and METLIN standards. Metabolite IDs were assigned to the corresponding ion of the two-dimensional data array.

### Tissue Lipid Extraction

Lipid extraction was conducted according to a modified Bligh/Dyer extraction method (Song et al., 2020). Samples were redissolved in isotopic mixed standards and then analyzed via Exion UPLC-QTRAP 6500 Plus (Sciex) with the electrospray ionization mode under the following conditions: curtain gas = 20; ion spray voltage = 5,500 V; temperature = 400°C; ion source gas 1 = 35; and ion source gas 2 = 35.

### Lipidomics Data Acquisition

Phenomenex Luna silica (3 μm, 1.5 mm × 200 mm) was selected as the chromatographic column. Lipids were extracted under A phase (chloroform/methanol/ammonia = 89.5:10:0.5) and B phase (chloroform/methanol/ammonia/water = 55:39:0.5:5.5). Extraction began with a 95% gradient of A phase from 0 to 5 min, then a linear decrease to 60% (in 7 min) for 4 min, a further decline to 30% for 15 min, and return to 95% for the last 5 min. Mass spectrometry multiple reaction monitoring was established for lipid identification and quantitative analysis (Lam et al., 2017, 2018).

### Metabonomics and Lipidomics Data Analysis

Metabonomics and lipidomics data were prepared and uploaded to the MetaboAnalyst software 4.0^2^ (Chong et al., 2019). Multivariate statistical analysis, cluster analysis, dimensionality reduction, and heatmaps were performed, based on the default parameters.

### Statistical Analysis

Statistical analyses were performed using GraphPad Prism software (version 8.0, GraphPad, San Diego, CA, United States). Statistically significant differences between the two groups were evaluated by two-tailed Student’s *t*-test. The relationships between lipid elements and cell types in the tumor microenvironment were analyzed by Pearson correlation analysis. A *p* < 0.05 was considered statistically significant. The area under the receiver operating characteristic (ROC) curve (AUC) was calculated to evaluate the accuracy of prediction.

## Results

### Transcriptome Analysis Reveals Changes in Lipid and Metabolic Pathways

**FIGURE 1:**
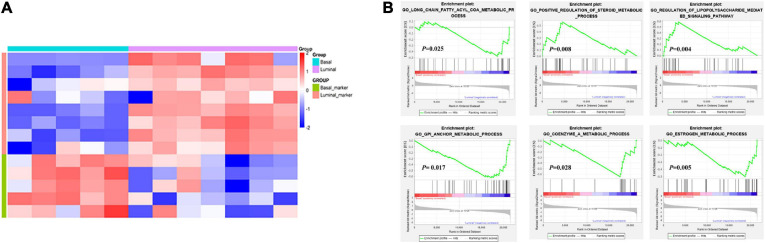
Transcriptome analysis reveals changes in lipid and metabolic pathways. **(A)** Expression heatmap of specific MIBC basal and luminal markers. **(B)** GSEA analysis showed the activation pathways in basal and luminal MIBC subtypes.

The establishment of tumor molecular subtypes has deepened our understanding of mutation gene profiles, tumor progression, and therapy responses (Robertson et al., 2018; Kamoun et al., 2020). Herein, we accurately classified 12 MIBC patients into basal and luminal subtypes using the BASE47 classifier based on transcriptome expression profiles (Damrauer et al., 2014). RNA-seq analysis revealed that basal and luminal MIBC subtype tumors displayed distinct gene expression patterns. Basal subtype had high levels of basal marker expression but low levels of luminal marker expression, while the luminal subtype displayed an opposite pattern (Figure 1A). GSEA analysis showed that activated long-chain fatty acyl-coA metabolic processes, positive regulation of steroid metabolic processes, and regulation of the lipopolysaccharide-mediated signaling pathway were associated with basal MIBC subtype, and glycosyl-phosphatidyl inositol (GPI) anchor metabolic process, coenzyme A metabolic process, and estrogen metabolic process were related to luminal MIBC subtype (Figure 1B). These results indicated that the lipid and metabolic pathways of basal and luminal MIBC subtypes were different.

### Distinct Lipid Profiles in Basal and Luminal MIBC Subtypes

**FIGURE 2:**
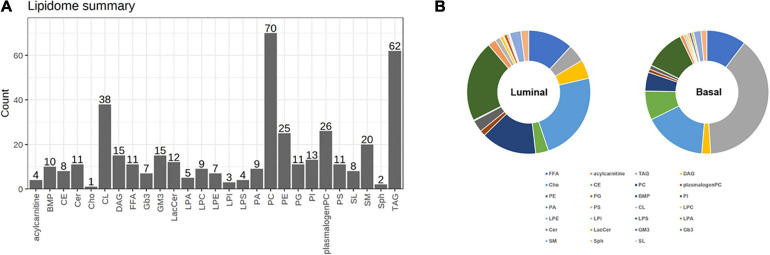
Distinct lipid profiles in basal and luminal MIBC subtypes. **(A)** The lipid types and amounts tested. **(B)** The relative frequencies of lipids in basal and luminal MIBC subtypes. BMP, bis (monoglycerol) phosphate ester; CE, cholesteryl esters; Cer, ceramides; Cho, free cholesterols; CL, cardiolipins; DAG, diacylglycerols; FFA, free fatty acids; Gb3, Ceramide trihexoside; GM3, monosialogangliosides; LacCer, lactosylceramides; LPA, lyso-PA; LPC, lyso-PC; LPE, lyso-PE; LPI, lyso-PI; LPS, lyso-PS; PA, phosphatidic acids; PC, phosphatidylcholines; PE, phosphatidylethanolamines; PG, phosphatidylglycerols; PI, phosphatidylinositols; PS, phosphatidylserines; SL, sulfatides; SM, sphingomyelins; Sph, sphingosine; TAG, triacylglycerols.

To further explore the differential lipids between basal and luminal MIBC subtypes. A total of 417 lipid elements could be qualitatively and quantitatively detected (Figure 2A). The content of lipid elements was significantly different in basal and luminal MIBC subtypes (Figure 2B). The differential lipid elements are shown in Table 1.

**TABLE 1:** Differential lipids of basal and luminal subtype (basal vs. luminal).

**Elevated lipids**	**Log2FC**	***p*-value**	**Declined lipids**	**Log2FC**	***p*-value**
SL d18:1/24:1h	3.9567	0.014*	CL68:6(16:1)	–2.3181	0.018*
SL d18:1/22:0	3.9406	0.012*	CL68:5(16:1)	–2.1704	0.045*
SL d18:1/24:0h	3.107	0.011*	SM d(18:1/26:0)	–1.9878	0.020*
SL d18:1/22:1	2.8944	0.012*	PC34:2 (16:1/18:1)	–1.8854	0.011*
SL d18:1/22:0h	2.8846	0.003*	PC34:1 (16:1/18:0)	–1.5558	0.009*
LacCer d18:1/14:0	2.2354	0.042*	BMP36:2	–1.5464	0.012*
GM3 d18:1/22:1	1.8766	0.002*	PI 34:1	–1.4499	0.034*
SM d18:1/20:1	1.6788	0.033*	BMP36:1	–1.4439	0.004*
SM d18:1/18:1	1.537	0.018*	CL70:7(16:1)	–1.3534	0.004*
SM d18:1/22:1	1.5258	0.045*	BMP36:4	–1.3515	0.040*
SM d18:1/18:0	1.4422	0.002*	CL70:6(16:1)	–1.3464	0.007*
SL	1.3674	0.013*	BMP	–1.2687	0.008*
SM d18:1/20:0	1.1882	0.000*	BMP36:3	–1.2364	0.042*
DAG38:4(18:0/20:4)	1.1665	0.004*	PC34:3 (16:1/18:2)	–1.2302	0.012*
FFA16:0	0.99879	0.000*	PA32:1	–1.0937	0.032*
FFA18:0	0.98576	0.000*	CL70:6(18:2)	–1.0884	0.033*
FFA	0.91768	0.000*	PC34:3	–1.0569	0.010*
LPI20:4	0.86724	0.004*	PG38:6	–0.98387	0.043*
SM d18:1/22:0	0.82901	0.005*	PC36:2	–0.82249	0.045*
PI 38:4	0.77328	0.025*	BMP38:4	–0.80758	0.022*
FFA18:1	0.75857	0.047*	PE38:6	–0.76824	0.043*
TAG52:5(16:0)	3.0945	0.0527	PE40:6	–0.74456	0.033*
Cer d(18:1/20:0)	2.8726	0.065368	CL66:4(16:1)	–2.2132	0.081798
LacCer d18:1/18:0	2.6487	0.082395	PC32:2 (16:1/16:1)	–1.8787	0.088617
GM3 d18:1/1:80	1.9886	0.070227	GM3 d18:0/26:0	–1.8627	0.070982
Cer d(18:1/14:0)	1.6358	0.066787	BMP34:1	–1.7599	0.057363
Gb3 d18:1/18:0	1.4349	0.098536	PE32:1	–1.6519	0.086111
GM3 d18:1/22:0	1.3553	0.053135	BMP34:2	–1.5235	0.051358
LysoPC18:0	1.0642	0.055778	CL70:5(16:1)	–1.3076	0.070479
FFA22:4	1.0201	0.092333	PC32:2	–1.2708	0.079861
FFA22:5	0.92609	0.087524	SM d18:1/25:0	–1.2471	0.052022
PA(36:1)	0.78922	0.066101	PC32:1	–1.2189	0.091632
SM d18:1/24:1	0.78035	0.056402	GM3 d18:0/25:0	–1.1178	0.058641
FFA20:4	0.75017	0.090839	PC36:2 (18:1/18:1)	–1.0772	0.050403
			PC36:3 (18:1/18:2)	–0.86314	0.090513
			LysoPC16:1	–0.84867	0.094135
			PC32:1 (16:0/16:1)	–0.84791	0.069625
			PC36:3	–0.78299	0.050465
			PC40:7 (22:6/18:1)	–0.69562	0.062649

* indicates *p* < 0.05.

### Potential Lipid Biomarkers of Basal and Luminal MIBC Subtypes

**FIGURE 3:**
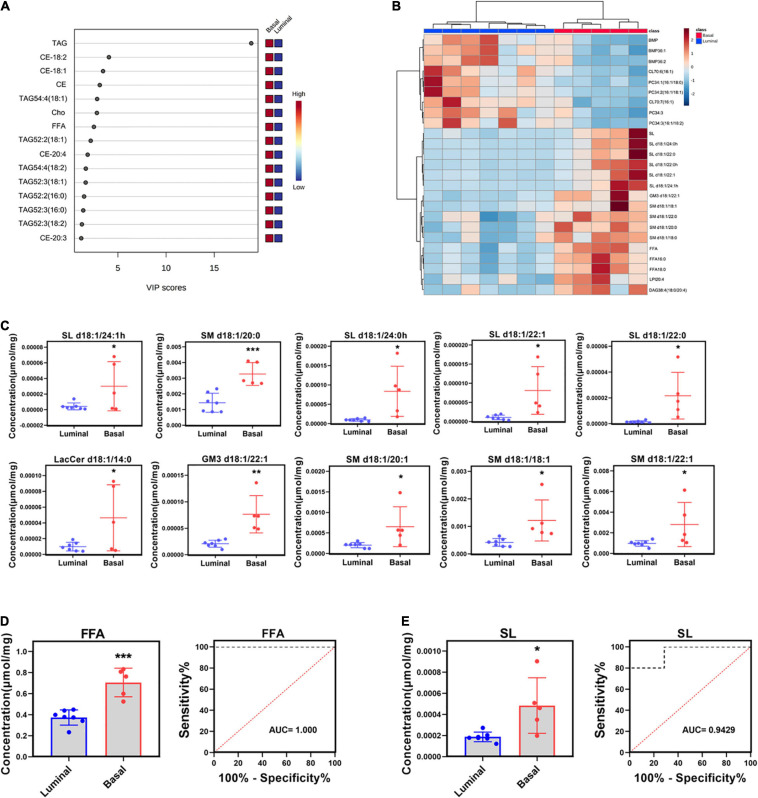
Potential lipid biomarkers of basal and luminal MIBC subtypes. **(A)** VIP score of altered lipid elements. **(B)** Heatmap of the top 25 altered lipid elements in basal and luminal MIBC subtypes. **(C)** The levels of the top 10 significantly differential lipid constituents in basal and luminal MIBC subtypes. **(D,E)** FFA and SL levels and AUC values. * indicates *p* < 0.05; ** indicates *p* < 0.01; and *** indicates *p* < 0.001.

Partial least squares discrimination analysis (PLS-DA) was performed to detect significant differential lipid elements between basal and luminal MIBC subtypes. By the variable import in project (VIP) score of each group, the top 15 lipid elements were identified (Figure 3A). The top 25 differential lipid elements between the basal and luminal MIBC subtypes are shown in Figure 3B. To explore the potential lipid biomarkers of basal and luminal MIBC subtypes, the following top 10 significantly differential lipid elements were analyzed: SL d18:1/24:1h, SM d18:1/20:0, SL d18:1/24:0h, SL d18:1/22:1, SL d18:1/22:0, LacCer d18:1/14:0, GM3 d18:1/22:1, SM d18:1/20:1, SM d18:1/18:1, and SM d18:1/22:1 (Figure 3C). Of these, SL d18:1/24:1h, SM d18:1/20:0, SL d18:1/24:0h, SL d18:1/22:1, SL d18:1/22:0, GM3 d18:1/22:1, SM d18:1/18:1, and SM d18:1/22:1 produced the highest AUC values (Supplementary Figure 1), indicating that these lipid elements could accurately separate basal and luminal MIBC subtypes, and these elements potentially to be targets of precision therapy in the future. In addition, the levels of total FFA and SL in the basal subtype were significantly higher than the luminal subtype, which displayed high AUC values (Figures 3D,E). These data indicated that SL d18:1/24:1h, SM d18:1/20:0, SL d18:1/24:0h, SL d18:1/22:1, SL d18:1/22:0, GM3 d18:1/22:1, SM d18:1/18:1, SM d18:1/22:1, FFA, and SL had potencies to be biomarkers for precisely distinguishing basal and luminal MIBC subtypes.

### Potential Lipid Biomarkers Are Associated With Tumor Microenvironment

**FIGURE 4:**
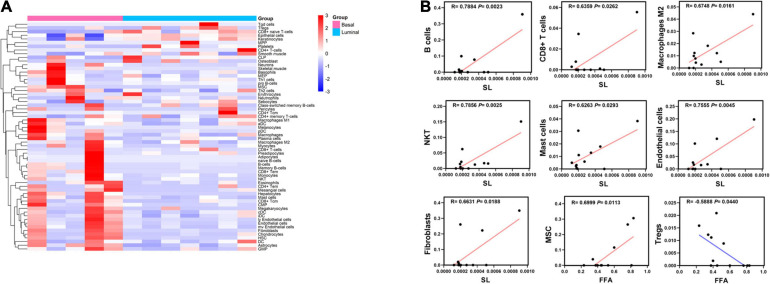
Potential lipid biomarkers are associated with tumor microenvironment. **(A)** Heatmap of the relative frequency of immune cell and stromal cell types in basal and luminal MIBC samples as identified by the “xCell” algorithm. Red line represents the maximum expression level and blue line represents the minimum expression level. **(B)** Pearson correlation analysis revealed the relationship among FFA, SL, immune, and stromal cell types. Red line represents the maximum expression level and blue line represents the minimum expression level.

Tumor microenvironment is composed of numerous cell types and greatly influences tumor progression and therapy response (Pfannstiel et al., 2019). We measured the relative frequencies of immune and stromal cell types using a new algorithm based on transcriptome profiles called “xCell” (Aran et al., 2017). The analysis showed that the relative frequencies of cell types in basal and luminal MIBC subtypes greatly differed (Figure 4A). Pearson correlation analysis showed that SL levels of samples were strongly related to B cells, CD8 + T cell, macrophages M2, natural killer T (NKT) cells, mast cells, endothelial cells, and fibroblasts values, while FFA levels of samples were closely related to mesenchymal stem cell (MSC) and regulatory T cell (Treg) values (Figure 4B). These data suggested that SL and FFA were both strongly associated with tumor microenvironment and may play key roles in MIBC progression.

### Distinct Metabolite Profiles in Basal and Luminal MIBC Subtypes

**FIGURE 5:**
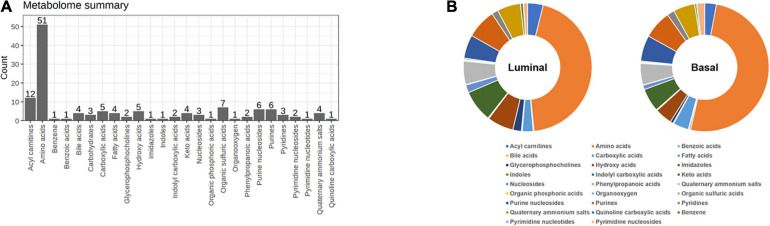
Distinct metabolite profiles in basal and luminal MIBC subtypes. **(A)** The types and amounts of metabolites examined in this study. **(B)** Relative frequencies of metabolites in basal and luminal MIBC subtypes.

To map the differential metabolite profile between basal and luminal MIBC subtypes, 133 metabolites were measured (Figure 5A). The metabolite profiles of basal differed from luminal MIBC subtypes (Figure 5B), and the differential metabolites are shown in Table 2.

**TABLE 2:** Differential metabolites of basal and luminal subtype (basal vs. luminal).

**Elevated metabolites**	**Log2FC**	***p*-value**	**Declined metabolites**	**Log2FC**	***p*-value**
Arabinonic acid	2.2107	0.000*	Glutathione	–1.964	0.025*
Allantoin	1.8753	0.032*	Oxidized glutathione	–1.6275	0.020*
Gamma-Glutamyl Glutamine	1.7797	0.000*	Glycerophos- phocholine	–1.6203	0.014*
Pyroglutamic acid	1.3859	0.002*	Butyrylcarnitine	–1.5932	0.001*
Glyceric acid	1.0352	0.033*	L-Malic acid	–1.1582	0.006*
Uridine	0.96311	0.031*	R-3-Hydroxybutyric acid	–1.145	0.025*
Uric acid	0.86495	0.022*	Propionylcarnitine	–1.01	0.023*
Glutaric acid	0.7218	0.016*	3′-AMP	–1.2204	0.052
tert-Butyl 3-amino-1,4,6,7-tetrahydro-5H-pyrazolo4,3-cpyridine-5-carboxylate	0.58832	0.014*	Pivaloylcarnitine	–2.2577	0.086
5-methoxy-L-tryptophan	2.2235	0.097	Xanthine	–1.3516	0.054
Methionine sulfoxide	1.3653	0.061	S-Glutathionyl-L-cysteine	–0.96333	0.096
N-Acetylleucine	1.3234	0.075	Leucyl-Serine	–0.79759	0.080
Taurodeoxycholic acid	0.97946	0.062	N-Acetyl-L-alanine	–0.74714	0.064
8-Hydroxy-deoxyguanosine	0.85958	0.065	Succinyla- denosine	–0.62835	0.062
Guanine	0.68097	0.074			

* indicates *p* < 0.05.

### Potential Metabolite Biomarkers of Basal and Luminal MIBC Subtypes

**FIGURE 6:**
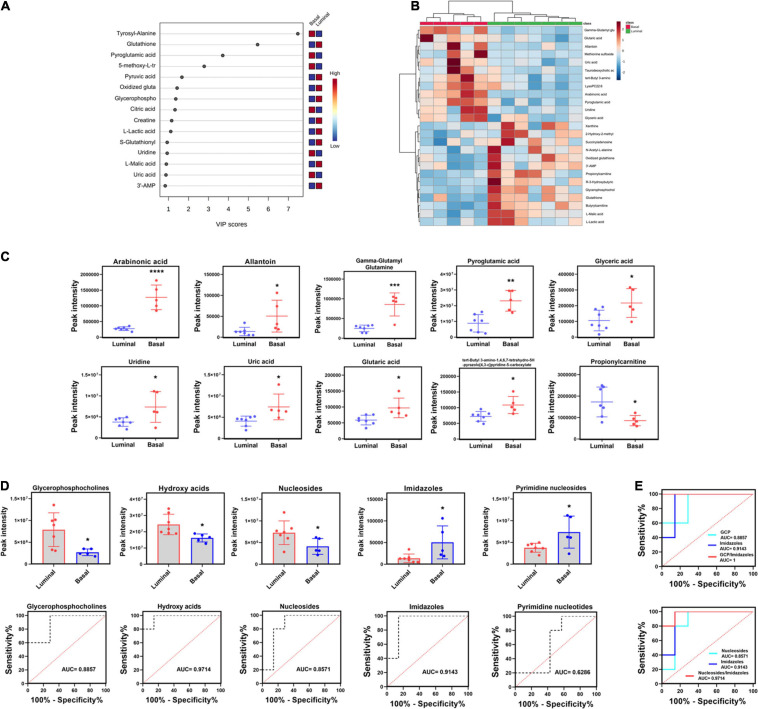
Potential metabolite biomarkers of basal and luminal MIBC subtypes. **(A)** VIP score of altered metabolites. **(B)** Heatmap of the top 25 altered metabolites in basal and luminal MIBC subtypes. **(C)** The peak intensity of the top 10 significantly differential metabolites in basal and luminal MIBC subtypes. **(D)** The peak intensity and AUC values of GCP, hydroxy acids, nucleosides, imidazoles, and pyrimidine nucleosides. **(E)** The AUC values of GCP/imidazoles and nucleosides/imidazoles ratios. * indicates *p* < 0.05; ** indicates *p* < 0.01; *** indicates *p* < 0.001; **** indicates *p* < 0.0001.

To further reveal the potential metabolite biomarkers in basal and luminal MIBC subtypes, we employed PLS-DA analysis to evaluate metabolite VIP scores. Based on the VIP score rank, the top 10 metabolites were identified: tyrosyl-alanine, pyroglutamic acid, 5-methoxy-L-tryptophan, citric acid, uridine, and uric acid were increased in the basal subtype, while glutathione, pyruvic acid, oxidized glutathione, glycerophosphocholine, creatine, L-lactic acid, S-glutathionyl-L-cysteine, L-malic acid, and 3′-adenosine monophosphate (3′-AMP) were increased in the luminal subtype (Figure 6A). The top 25 differential metabolites are shown in Figure 6B. The peak intensities of the top 10 significantly different metabolites in basal and luminal MIBC subtypes are shown in Figure 6C. To further identify potential metabolite biomarkers in basal and luminal MIBC subtypes, we analyzed the levels of the main types of metabolites. It was found that the levels of glycerophosphocholine (GCP), hydroxy acids, and nucleosides increased in the luminal subtype, while the levels of imidazoles and pyrimidine nucleoside were higher in the basal than in the luminal subtype. These metabolites presented different AUC values (Figure 6D). Remarkably, the ratios of GCP/imidazoles (AUC = 1) and nucleosides/imidazoles (AUC = 0.9714) had higher AUC values than GCP (AUC = 0.8857), nucleosides (AUC = 0.8571), or imidazoles (AUC = 0.9143) levels alone (Figure 6E). The above results indicated that the ratios of GCP/imidazoles and nucleosides/imidazoles had a greater capacity to differentiate basal and luminal MIBC subtypes than the single metabolites; these ratios could be used as potential biomarkers to distinguish basal and luminal MIBC subtypes.

## Discussion

Muscle invasive bladder cancer is a molecularly heterogeneous disease with high recurrence rates and poor prognosis (Prasad et al., 2011; Meeks et al., 2020). The BASE47 classifier divides MIBC into basal and luminal subtypes based on transcriptome expression profiles. The differentiation pattern, histological characteristic, overall survival, and therapy response of basal and luminal MIBC subtypes are significantly different (Kamoun et al., 2020). This classifier provides a new framework for studying MIBC heterogeneity and has potential values for clinical application (Ochoa et al., 2016; Fong et al., 2020). Metabolic reprogramming of tumors drives tumor progression by many aspects (Pavlova and Thompson, 2016). Although previous studies have explored the metabolic profile and identified metabolites associated with recurrence and poor prognosis of BC (Armitage and Ciborowski, 2017; Loras et al., 2018; Zhang et al., 2018), the differential lipids and metabolites between basal and luminal MIBC subtypes remain unclear. Knowledge of these profiles may provide potential biomarkers and therapy targets for clinical application. In this study, we integrated transcriptomics, lipidomics, and metabolomics analysis to reveal the differential lipid and metabolite profiles between basal and luminal MIBC subtypes, providing potential lipid and metabolite biomarkers for precision therapy of MIBC.

According to the BASE47 classifier, we divided MIBC patients into basal and luminal subtypes based on transcriptomic expression profiles. RNA-sequencing analysis revealed that the lipid and metabolic pathways of basal and luminal MIBC subtypes differed significantly, which suggested that basal and luminal MIBC subtype potentially underwent lipid and metabolic reprogramming (Lee et al., 2018). To further explore the lipid profiles of basal and luminal MIBC subtypes, we evaluated 417 tissue lipid elements in basal and luminal MIBC subtypes. Results showed that there were significant differences in the lipid profiles of basal and luminal MIBC subtypes. The top 10 differential lipid elements were enriched in the basal subtype, eight of which exhibited maximum AUC values and could be considered as potential biomarkers: SL d18:1/24:1h, SM d18:1/20:0, SL d18:1/24:0h, SL d18:1/22:1, SL d18:1/22:0, GM3 d18:1/22:1, SM d18:1/18:1, and SM d18:1/22:1. Due to the small sample size, the differential lipids identified between basal and luminal MIBC subtypes may be limited. Thus, in our cohort, the top 10 differential lipids between basal and luminal MIBC subtypes were all enriched in basal MIBC subtype. Furthermore, examination of the main types of lipids revealed that the total FFA and SL levels of the basal samples were higher than that of the luminal samples. These lipids display strong potencies to be biomarkers. Additionally, according to a new algorithm, we inferred the relative frequencies of immune and stromal cells in samples based on their mRNA profiles. Pearson correlation analysis showed that FFA and SL were significantly related to specific immune and stromal cell types in the tumor microenvironment. Indeed, FFA drives tumor progression by stimulating cancer cell proliferation and promotes CD8 + TRM cells to persist in tumor tissue to mediate protective immunity (Iwamoto et al., 2018; Zhang et al., 2020). Meanwhile, SL is involved in cancer progression and improves sensitivity of tumor cells to microenvironmental stress factors including hypoxia and anticancer drugs (Suchanski and Ugorski, 2016; Suchanski et al., 2018). Therefore, FFA and SL may play important roles in MIBC progression and potentially used to be biomarkers of basal and luminal MIBC subtypes.

During tumor reprogramming, metabolic patterns of cancer cells are changed to adapt to the new microenvironments, which makes it important to deeply understand cancer metabolic profiles (Kim and DeBerardinis, 2019; La Vecchia and Sebastian, 2020). To reveal the differential metabolite profiles between basal and luminal MIBC subtypes, we evaluated a total of 133 metabolites. Our results suggested that GCP, hydroxy acids, nucleosides, imidazoles, and pyrimidine nucleosides could accurately distinguish the basal subtype from the luminal subtype. Furthermore, the AUCs of the GCP/imidazoles and nucleosides/imidazoles ratios were higher than those of GCP, nucleosides, and imidazoles alone, suggesting that these ratios were more sensitive for distinguishing basal from luminal MIBC subtypes. According to previous reports, GCP, nucleosides, and imidazoles drive cancer progression; they are associated with poor prognosis of several types of cancer (Moestue et al., 2012; Dolinar et al., 2018; Long and Wang, 2019). Therefore, the GCP/imidazoles and nucleosides/imidazoles ratios have potential clinical applications as biomarkers, while GCP, nucleosides, and imidazoles may be the targets of MIBC precision therapy.

The occurrence and development of tumor is a complex process, which is coregulated by genomics, epigenomics, transcriptomics, proteomics, metabolomics, microbiome, and other factors (Menyhart and Gyorffy, 2021). Single omics studies cannot fully reveal the characteristics of tumors and provide reliable biomarkers. In this study, the integration of transcriptomics, lipidomics, and metabonomics can be used to develop subtype-specific biomarkers and therapeutic targets and may provide more precise predictions for disease progression and prognosis. However, it should be noted that this study has some limitations. First, the sample size was small. Additional larger and independent cohorts should be analyzed to reveal more valuable lipidomic and metabonomic biomarkers. Second, this study only explored the differential lipid and metabolite profiles between basal and luminal MIBC subtypes. The accuracy and sensitivity of the potential biomarkers identified here needed to be confirmed in larger cohorts. Third, there is no strict exclusion to some potential conditions that influence lipid and metabolite profiles from our analysis, such as diabetes and hyperlipemia.

In conclusion, our study integrated transcriptomic, lipidomic, and metabolomic analysis to reveal the differential lipid and metabolite profiles between basal and luminal MIBC subtypes. It was also found that FFA, SL, the GCP/imidazoles, and nucleosides/imidazoles ratios have strong potencies to be biomarkers for distinguishing basal from luminal MIBC subtypes.

## Data Availability Statement

All the raw data used in this manuscript will be made available to any qualified researcher without reservation. The data presented in the study are deposited in the GEO repository, accession number: GSE179440.

## Ethics Statement

The studies involving human participants were reviewed and approved by the Ethics Committee of The First Affiliated Hospital of Guangxi Medical University. The patients/participants provided their written informed consent to participate in this study.

## Author Contributions

TL, QW, and ZT designed the study. CF, LP, and ST wrote the manuscript. LH, XW, YT, YX, and ZL analyzed the results. All authors contributed to the manuscript and approved the submitted version.

## Conflict of Interest

The authors declare that the research was conducted in the absence of any commercial or financial relationships that could be construed as a potential conflict of interest.

## Publisher’s Note

All claims expressed in this article are solely those of the authors and do not necessarily represent those of their affiliated organizations, or those of the publisher, the editors and the reviewers. Any product that may be evaluated in this article, or claim that may be made by its manufacturer, is not guaranteed or endorsed by the publisher.

## Footnotes

1
http://xcell.ucsf.edu/

2
www.metaboanalyst.ca

## Supplementary Material

Supplementary Figure 1 The AUC of eight lipids with differential distributions in basal and luminal MIBC subtypes. The AUC values of SL d18:1/24:1h, SM d18:1/20:0, SL d18:1/24:0h, SL d18:1/22:1, SL d18:1/22:0, GM3 d18:1/22:1, SM d18:1/18:1, and SM d18:1/22:1.
