# Supplementary figures and images for: Integrative Transcriptomic, Lipidomic, and Metabolomic Analysis Reveals Potential Biomarkers of Basal and Luminal Muscle Invasive Bladder Cancer Subtypes
Source: Front Genet. 2021 Aug 16;12:695662. doi: 10.3389/fgene.2021.695662 (PMC8415304; doi:10.3389/fgene.2021.695662)

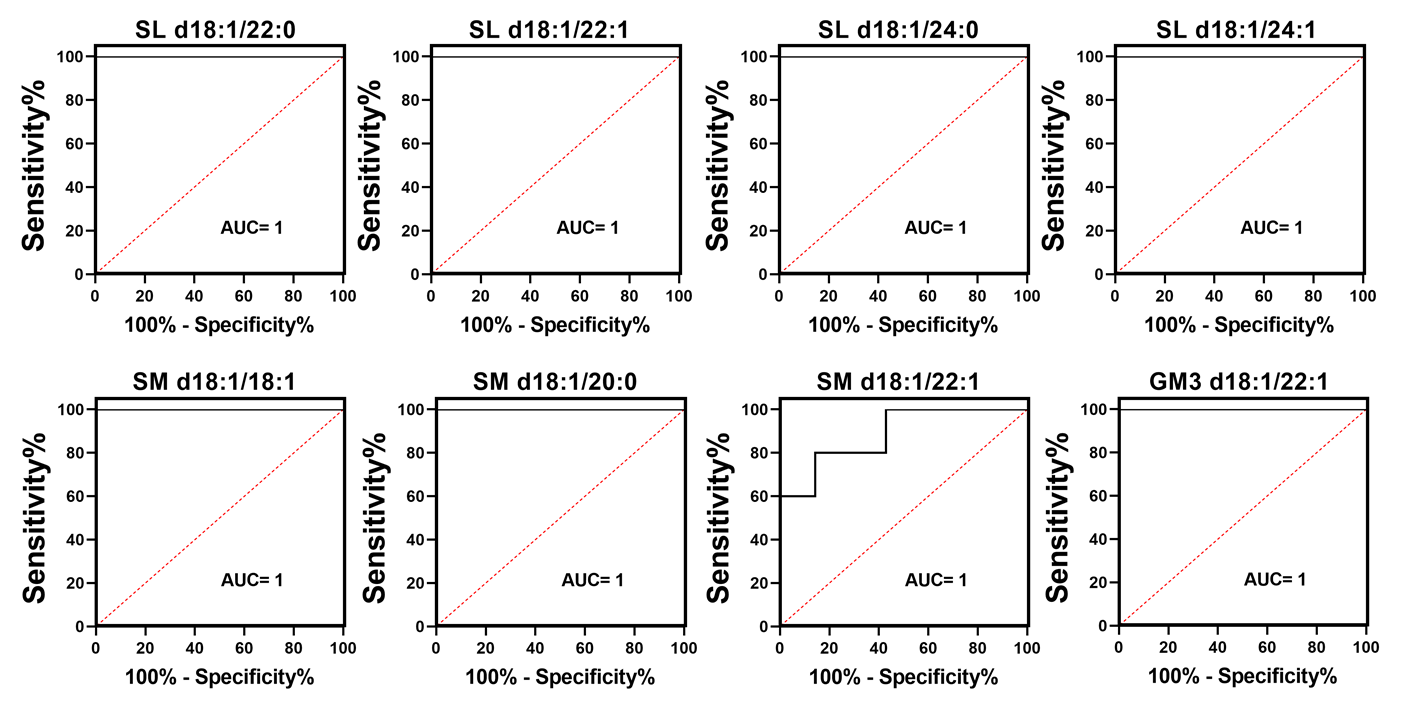

Supplement: Supplementary Figure 1 — The AUC of eight lipids with differential distributions in basal and luminal MIBC subtypes. The AUC values of SL d18:1/24:1h, SM d18:1/20:0, SL d18:1/24:0h, SL d18:1/22:1, SL d18:1/22:0, GM3 d18:1/22:1, SM d18:1/18:1, and SM d18:1/22:1. [file Image_1.TIFF]
